# Preattentive Extraction of Abstract Auditory Rules in Speech Sound Stream: A Mismatch Negativity Study Using Lexical Tones

**DOI:** 10.1371/journal.pone.0030027

**Published:** 2012-01-06

**Authors:** Xiao-Dong Wang, Feng Gu, Kang He, Ling-Hui Chen, Lin Chen

**Affiliations:** 1 CAS Key Laboratory of Brain Function and Diseases, School of Life Sciences, University of Science and Technology of China, Hefei, China; 2 Auditory Research Laboratory, University of Science and Technology of China, Hefei, China; 3 iFlytek Speech Laboratory, School of Information Science and Technology, University of Science and Technology of China, Hefei, China; University of Salamanca- Institute for Neuroscience of Castille and Leon and Medical School, Spain

## Abstract

**Background:**

Extraction of linguistically relevant auditory features is critical for speech comprehension in complex auditory environments, in which the relationships between acoustic stimuli are often abstract and constant while the stimuli per se are varying. These relationships are referred to as the abstract auditory rule in speech and have been investigated for their underlying neural mechanisms at an attentive stage. However, the issue of whether or not there is a sensory intelligence that enables one to automatically encode abstract auditory rules in speech at a preattentive stage has not yet been thoroughly addressed.

**Methodology/Principal Findings:**

We chose Chinese lexical tones for the current study because they help to define word meaning and hence facilitate the fabrication of an abstract auditory rule in a speech sound stream. We continuously presented native Chinese speakers with Chinese vowels differing in formant, intensity, and level of pitch to construct a complex and varying auditory stream. In this stream, most of the sounds shared flat lexical tones to form an embedded abstract auditory rule. Occasionally the rule was randomly violated by those with a rising or falling lexical tone. The results showed that the violation of the abstract auditory rule of lexical tones evoked a robust preattentive auditory response, as revealed by whole-head electrical recordings of the mismatch negativity (MMN), though none of the subjects acquired explicit knowledge of the rule or became aware of the violation.

**Conclusions/Significance:**

Our results demonstrate that there is an auditory sensory intelligence in the perception of Chinese lexical tones. The existence of this intelligence suggests that the humans can automatically extract abstract auditory rules in speech at a preattentive stage to ensure speech communication in complex and noisy auditory environments without drawing on conscious resources.

## Introduction

The encoding of abstract rules, which are abstract representations of knowledge in memory, is a critical cognitive function, essential for perception of the world [1]. It is a central structure in cognitive science [2]. For biological organisms, the capacity to extract abstract rules from complex environments is beneficial for survival [3]. For humans, this capacity may be relevant to everyday speech communication because often listeners are confronted with considerable variation in intensity and spectral-temporal property of speech signals, in addition to ambient noise [4], [5]. To cope with this variability, it is necessary for the auditory cortex to process the relationships between the stimuli to derive and form efficient abstract representations, such as speaker identity and speech content [6]. These relationships, which are referred to as abstract auditory rules in speech, are abstract and constant, while the stimuli per se are varying.

Current studies largely focus on the neural mechanisms of encoding abstract auditory rules in speech sounds at an attentive stage, including top-down modulation using functional magnetic resonance imaging (fMRI) techniques. For example, some studies have explored the learning of an abstract representation of vowels by using a linear support-vector-machine learning approach [7], [8]. There are also fMRI studies indicating that the process of encoding abstract auditory rules in speech is associated with the supramarginal gyrus (SMG), which has often been interpreted as evidence that the SMG has a critical role in the working memory of phonological information [9], [10], [11]. Evidence from the fMRI studies suggests that there may exist an auditory sensory intelligence for the extraction of abstract auditory rules in speech because the encoding of these rules is also associated with lower-level auditory regions (Heschl's gyrus) [8].

An effective approach to isolating the brain response component contributed by the cognitive processing at an early stage, or at a preattentive stage, with a sufficient temporal resolution is essential to determining whether the brain has a sensory intelligence for extracting abstract auditory rules in speech. Neuroimaging techniques have excellent spatial resolution but measure hemodynamic responses with a low temporal resolution (from seconds to tens of seconds) [12]. As such, they are not considered appropriate for this purpose. Observations made from neuroimaging studies, which require the execution of an abstraction task (such as the extraction of abstract auditory rules in speech), may estimate the temporally aggregated neural events including those at an attentive stage. To this end, the mismatch negativity (MMN) can be an efficient tool for investigation of preattentive extraction of abstract auditory rules [13], [14]. The MMN is a powerful tool for study of the automatic processing of auditory linguistic inputs [15], [16], [17], [18], [19]. The MMN usually peaks 100–250 ms after onset of stimulus and is an index of the brain's sensory intelligence in the preattentive encoding of abstract rules in audition [20].

There are a number of studies available addressing the human ability of preattentive extraction of abstract auditory rules in the non-speech domain [21], [22], [23], [24]. In these studies, varying sinusoidal tones were typically used to form an auditory stream with an abstract auditory rule embedded in it. In a study by Saarinen *et al*., for instance, the stimuli were a stream of tone pairs differing in absolute frequency levels to construct a varying auditory stream [22]. In the stream, most of the pairs were made up of stimuli that shared the same direction (rising or falling) of the within-pair frequency change to form an embedded abstract auditory rule. Occasionally, this rule was violated with random occurrence of pairs whose changes in frequency were in opposite directions. They found that this violation of the embedded rule elicited an MMN response, indicating that the human auditory system is able to encode not only the physical features of repetitive stimuli, but also abstract attributes (direction of frequency change) from a set of individual varying physical events at a preattentive stage.

Although previous studies in the non-speech domain suggest that the preattentive extraction of abstract auditory rules in pitch patterns is critical to the human perception of music and comprehension of spoken language, sparse information is available about the utilization of this ability directly in the speech domain. For non-tonal languages, there has been evidence showing that MMNs can be elicited preattentively by categorical (abstract) violations of phoneme information in speech sound streams [25], [26], [27], [28]. It is still not well-addressed whether or not there is a sensory intelligence that enables one to automatically encode implicit and abstract auditory rules of pitch patterns in constantly changing speech streams at a preattentive stage. The challenge in addressing this issue is fabricating a complex auditory stream of speech with abstract rules embedded in it. Mandarin Chinese, which uses lexical tones in addition to consonants and vowels to signal word meaning, provides an easy solution. In the present study, we used lexical tones to form a complex auditory stream containing an abstract auditory rule in linguistic pitch patterns. We deployed Mandarin Chinese vowels differing in formant, intensity, and the level of pitch per se to form a complex and varying auditory stream in which most of sounds shared flat lexical tones to form an embedded abstract auditory rule. Occasionally, this rule was violated by a random occurrence of those with rising or falling lexical tones. The extraction of this rule at a preattentive stage was measured with whole-head recordings of the MMN.

## Results

### Standard stimuli evoked a robust P1-N1-P2 complex

**Figure 1:**
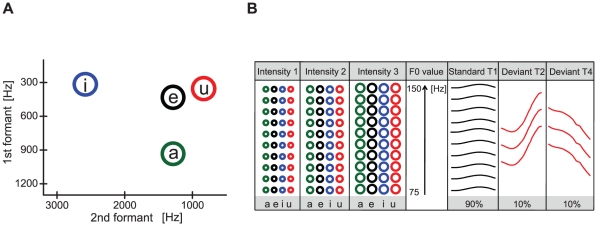
Stimuli and experimental design. (A) Synthesized vowel stimuli/a/, /e/, /i/, and/u/in the F1–F2 space. (B) The standard stimuli shared the relative flat pitch contour but differed in formant, intensity, and level of pitch. There were 120 different combinations of standard stimuli (4 vowels * 3 intensities * 10 pitch levels). Two deviant types were defined, the rising deviant (T2) and the falling deviant (T4). Within each deviant type, the stimuli differed in formant, intensity, and level of pitch. There were 36 different combinations of stimuli (4 vowels * 3 intensities * 3 pitch levels) for each deviant type.

**Figure 2:**
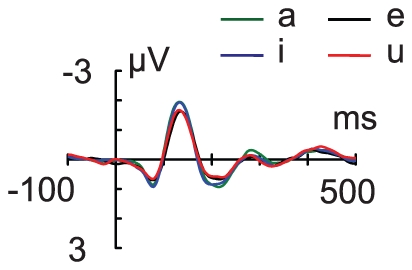
Grand average of event-related potential (ERP) waveforms in response to the standard stimuli. The ERP responses to the standard stimuli/a/, /e/, /i/, and/u/at the Fz electrode were fractionated. A robust P1-N1-P2 complex was recorded for all the vowels.

Native Chinese speakers were continuously presented with Chinese vowels/a/, /e/, /i/, or/u/ (Figure 1A) differing in formant, intensity, and level of pitch (Figure 1B) to form a complex and varying auditory stream. In this stream, most of sounds shared flat lexical tone (T1) (standard stimuli) to form an embedded abstract auditory rule. Occasionally, this rule was violated by a random occurrence of those with a rising (T2) or falling (T4) lexical tone (deviant stimuli). A robust event-related potential (ERP) in response to the standard stimuli could be recorded. The ERP in response to either of the four vowels in the standard stimuli had a P1-N1-P2 complex (Figure 2). The robust N1 responses to all standards indicate minimal N1 adaptation, partially due to the varying ongoing auditory streams.

### Violation of abstract auditory rules of pitch evoked an MMN response without subsequent P3a

**Figure 3:**
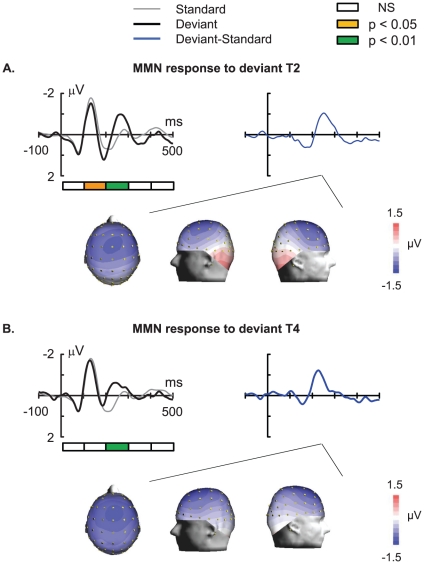
Grand average waveform and topographical map of mismatch negativity (MMN). MMN was derived by subtracting the ERP response to the standard from that to the deviant stimuli. The topographical maps were constructed at the peak latency of the MMN. (A) ERP waveforms at the Fz electrode for the rising deviant type (T2). (B) ERP waveforms at the Fz electrode for the falling deviant type (T4).

The MMN is an index of early auditory cognitive processing [29], [30]. The ERP P3a component is an index of involuntary switch of attention [31], [32]. To detect the presence of MMN and P3a components, we defined five time windows (0–100 ms, 100–200 ms, 200–300 ms, 300–400 ms, and 400–500 ms) (Figure 3) to calculate the mean amplitude of the standard ERP and deviant ERP from the Fz electrode for each subject. Significant differences between the ERP evoked by standard stimuli (T1) and the ERP evoked by the rising tone deviant (T2) were found in only two time windows: 100–200 ms and 200–300 ms [F(1, 12) = 7.3, *P*<0.05 and F(1, 12) = 34.4, *P* = 0.0001 respectively], indicating the absence of P3a component and the presence of a robust MMN elicited by violation of abstract auditory rules due to the random occurrence of T2 (Figure 3A). Significant differences in the ERP evoked by standard stimuli (T1) and the ERP evoked by the falling tone deviant (T4) were found only at 200–300 ms [F(1,12) = 10.8, *P*<0.01], indicating the absence of P3a component and the presence of a robust MMN elicited by violation of the abstract auditory rules due to the random occurrence of T4 (Figure 3B). Analysis of waveforms recorded from the fronto-central site (FCz), central site (Cz), and parietal site (Pz) also showed an MMN response without the a P3a component (Figure S1). The absence of P3a component indicates that the violation of the abstract rule did not arouse an involuntary switch of attention of the subjects.

The scalp topographic maps of MMN responses at the peak latencies showed fronto-central distributions for both types of deviants (Figure 3). The statistical analysis of main effect of factor sagittal electrode site showed the response to be more focal and restricted toward the frontal sites (F(2, 24) = 17.354, *P* = 0.001). The statistical analysis of the main effect of the lateral electrode site showed the response to be more focal and restricted toward the central sites (F(2, 24) = 4.536, *P* = 0.037). Post-hoc testing using the Tukey's test confirmed the absence of significant differences between mean amplitudes at electrode positions over the left and right hemispheres. Average dipole strengths obtained from dipole solutions for individual subjects did not show any hemisphere laterality in the response (Figure S2). No significant effect on either lexical tone (F(1, 11) = 1.53738, *P* = 0.24081) or hemisphere (F(1, 11) = 0.08131, *P* = 0.78082) or interactions between the two factors (F(1, 11) = 0.20744, *P* = 0.65764) was detected by dipole analysis.

### Relationship between behavioral reaction time (RT) and MMN peak latency

**Figure 4:**
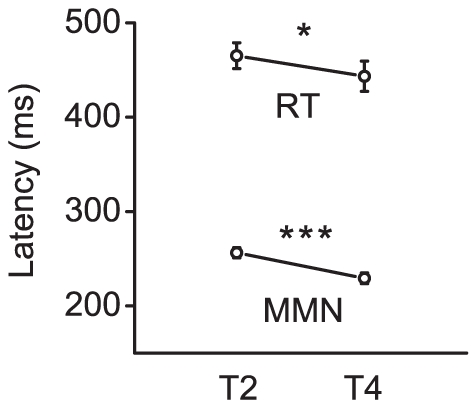
MMN peak latency and behavioral reaction time (RT). The time of preattentive detection of abstract rule violations for the two deviant types (T2 and T4) was reflected as the peak latency of MMN. After the subjects learned the rule and completed behavioral training, their behavioral RTs for detecting the abstract rule violations were recorded. Vertical bars indicate the standard error of the mean.

During acquisition of electroencephalogram (EEG) data, subjects neither acquired explicit knowledge of the abstract auditory rules nor became aware of the violations of those rules. After acquisition of EEG data, subjects were behaviorally trained to detect the violations of the abstract rules. The subjects all achieved high hit rates for detecting the rising deviant (T2) and the falling deviant (T4), with the former marginally lower than the latter [89.8±3.9% (s.e.m.) vs. 92.6±3.2% (s.e.m.), F(1,12) = 3.990, *P* = 0.069]. The RT for detecting the rising deviant was significantly longer than the falling deviant [465.2±13.8 ms (s.e.m.) vs. 443.4±16.0 ms (s.e.m.), F(1, 12) = 8.352, *P* = 0.014] (Figure 4). In parallel, the MMN peak latency for the rising deviant was also longer than that of the falling deviant [254.1±4.6 ms (s.e.m.) vs. 227.7±5.3 ms (s.e.m.), F(1, 12) = 35.0, *P* = 0.0001] (Figure 4). The parallel between the behavioral RT and the MMN peak latency suggests that the preattentive extraction of abstract auditory rules of lexical tones predicts and facilitates perception at an attentive stage.

## Discussion

In the present study, we used lexical tones to form a complex and varying auditory stream of speech sounds containing an abstract auditory rule. Our experiments demonstrated that there is an auditory sensory intelligence in preattentive perception of Chinese lexical tones. The existence of this intelligence, as revealed by the presence of MMN, indicates that the human can extract abstract auditory rules from speech streams automatically. Although some evidence is already available to show that humans can extract auditory rules in pitch patterns in the non-speech domain [21], [22], [23], [24], our results clearly indicate that this is also true in the speech domain. We suggest that this capacity may help humans to understand speech in complex and noisy auditory environments without drawing on conscious resources.

Our study demonstrates that the extraction of abstract auditory rules of lexical tones is automatic and preattentive. This conclusion is supported by the following experimental evidence. First, the MMN was recorded passively, as shown by the fact that the subjects were instructed to ignore the sound stimuli and focus their attention on a self-selected silent movie. Second, the absence of P3a component after MMN (Figure 3) indicates that violation of the abstract auditory rules does not arouse an involuntary switch of attention. The presence of the P3a component is believed to be an index of involuntary switch of attention [31], [32], [33], [34]. Finally, following the EEG recording, none of the subjects obtained explicit knowledge of the abstract rule or became aware of the violation of the rule, suggesting that the extraction of the rule is fully automatic and preattentive.

Our results suggest that the preattentive encoding of abstract auditory rules of lexical tones can predict perception during a later attentive stage. In the present study, the MMN in response to the falling deviant (T4) peaked significantly earlier than that in response to the rising deviant (T2) (Figure 4). Interestingly, the attentive behavioral RT for detection of the violations of the abstract rules occurred parallel to preattentive MMN peak latency: The RT for the falling deviant (T4) is significantly shorter than that in response to the rising deviant (T2). This is consistent with previous behavioral dichotic listening studies that demonstrated that the falling lexical tone (T4) is easier to perceive than the rising lexical tone (T2) [35], [36]. One possible explanation is that the neurons of the auditory cortex exhibit different detection sensitivities to rising and falling lexical tones. However, the opposite is true for the sinusoidal tones: the brain response to increases in this frequency is stronger than to decreases [37], [38]. This suggests that the brain processes speech pitch patterns and non-speech pitch patterns in different ways. In the present study, the parallel between the behavioral RT and the MMN peak latency indicates that the attentive detection of the violation of abstract auditory rules is facilitated by the sensory intelligence at a preattentive stage. This is consistent with the findings of an earlier report which demonstrated that conscious novelty detection in humans is governed by preattentive sensory memory [39].

We are not certain whether the conclusions drawn from the present study can be generalized to all humans (including speakers of non-tonal languages), or even to the non-human species (as evolutionary precursors). What would have happened if the present experiment had been performed in the non-tonal language speakers? If the auditory processing of lexical tones at an early preattentive stage is shaped mainly by the acoustic properties rather than the linguistic status [40], [41], we suspect that the subjects would still have been able to extract some rules from the auditory stream at a preattentive stage. In this case, it is very likely that these speakers would extract the rule as an abstract auditory rule in pitch patterns rather than as that in lexical tones. This is because the abstract auditory rules constructed with lexical tones embedded in the speech sound stream are linguistically meaningless to speakers of non-tonal languages (Assume the pitch contour is not used by the non-tonal language speaker to encode intonation differences). If the auditory processing of lexical tones at an early preattentive stage is primarily determined by the linguistic function of the lexical tones, we suspect that the subjects would not extract any rules from the auditory stream at a preattentive stage. Under any circumstances, it is unlikely that long-term memory associated with speech is triggered in speakers of non-tonal languages when they extract abstract auditory rules constructed with including lexical tones in the speech stream.

In summary, our study demonstrates that there is auditory sensory intelligence involved in the perception of Chinese lexical tones. The existence of this intelligence indicates that the human beings can extract abstract auditory rules in speech stream at a preattentive stage already to ensure speech communication in a complex environment. Furthermore, this sensory intelligence is separated from the subsequent cognitive process of involuntary attention switching, indicating that the central auditory system is able to process a large part of its input automatically and preattentively, even in complex auditory environments, without requiring or arousing the limited resources of the controlled processing system. The automatic sensory intelligence in audition forms the basis for high-order cognitive processes [14].

## Materials and Methods

### Subjects

Thirteen native speakers of Mandarin Chinese (6 males and 7 females) with no history of neurological or psychiatric impairment participated in the present study. The subjects were 21–28 years old, musically untrained, and all students at the University of Science and Technology of China. All of the subjects reported normal hearing and were right-handed according to an assessment with the Chinese version of the Edinburgh Handedness Inventory [42]. They were compensated for their participation. They were recruited for EEG recordings and participated in the behavioral test about 1 hour following the EEG recordings. The protocols and experimental procedures deployed in the present study were reviewed and approved by the Biomedical Research Ethics Committee of the University of Science and Technology of China. All subjects provided written informed consent.

### Stimuli

We used an auditory odd-ball sequence to construct a complex and varying auditory stream in which an abstract auditory rule in speech was embedded. All stimuli used in the auditory stream were synthesized Mandarin Chinese vowels/a/, /e/, /i/, and/u/(Figure 1A). The materials used for synthesis of vowels were originally pronounced by an adult male speaker (Sinica Corpus, Institute of Linguistics, Chinese Academy of Social Sciences, Beijing, China). Synthesis parameters for voice fundamental frequency (F0) and vowel duration were similar to those reported by a previous study [43]. The speech waveform was generated by the high-quality speech synthesizer STRAIGHT (Speech Transformation and Representation using Adaptive Interpolation of weiGHTed spectrum) using a source-filter model [44]. A periodical excitation sequence was used to stimulate the vocal tract filter to produce vowels. Line spectral pairs (LSPs) were used to model the vocal tract filter [45]. The LSP parameters were extracted using frame-by-frame linear predictive coding (LPC) [46]. In order to keep the formant structure stable, we selected representative LSP parameters from the extracted LSPs for all the frames of the vowels during the synthesis process. In this way, the only difference between the same generated vowels came from the F0. A time domain short waveform was generated and overlap-added at each pitch-synchronous point to generate speech waveform using time-domain pitch-synchronous overlap-add techniques (TD-PSOLA) [47]. Vowels/a/, /e/, /i/, and/u/presented with the flat tone (tone 1) were synthesized with three overall degrees of intensity with 3 dB attenuation and 10 levels of pitch (from 78 Hz to 150 Hz, the interval for the adjacent two pitch levels was 8 Hz) (Figure 1B). Standard stimuli were the vowels sharing the same flat pitch contour (T1), which had 120 different sound combinations (4 vowels * 3 intensities * 10 pitch levels). The deviant stimuli were vowels/a/, /e/, /i/, and/u/, synthesized with rising (T2) and falling tones (T4) with three degrees of intensity (3 dB attenuation each) and three levels of pitch (Figure 1B). Hence, there were two types of deviant stimuli, rising tone deviant stimuli (T2) and falling tone deviant stimuli (T4). Each deviant group contained 36 different sound combinations (4 vowels * 3 intensities * 3 pitch levels). For the four vowel types, the first formant (F1) and second formant (F2) were 930 Hz/1310 Hz for/a/, 400 Hz/1300 Hz for/e/, 300 Hz/2700 Hz for/i/, and 320 Hz/980 Hz for/u/. All stimuli were 150 ms in duration with 5 ms of linear rise and fall time. The synthesized stimuli were typical representatives of Chinese lexical tones. After synthesis, the stimuli were recognized by 10 native Chinese speakers (4 males and 6 females) who were not included in the following EEG and behavioral experiments. All of them reported that the stimuli were good exemplars of Chinese lexical tones.

### Procedure

The stimuli used in this experiment differed in formant, intensity, and level of pitch. They were presented in odd-ball blocks so as to form a complex auditory stream. In this stream, stimuli that shared the flat tone (T1) served as the standard stimuli, while those that shared the rising tone (T2) and falling tone (T4) formed the two types of deviant stimuli. Within each odd-ball block only one deviant type was presented, with a probability of 10%. The standards were presented with a probability of 90%. For each subject, four blocks were presented (two blocks for each type of deviant). For each type of deviant, a total of 3600 stimuli (3240 standards and 360 deviants) were presented.

MMN was recorded with the contrast of the relative pitch contour between the standards and deviants. During the EEG experiment, the subject was instructed to ignore the auditory stimuli and watch a silent movie with subtitles. Each stimulus was presented diotically at 62, 65, and 68 dB SPL through headphones (TDH-39; Telephonics, Farmingdale, NY, U.S.) in an electrically shielded, soundproof room with a stimulus onset asynchrony (SOA) of 700 ms. A behavioral study was conducted about 1 hour after the ERP recording of each subject. After the EEG recording, subjects were informed of the rule (relative pitch contour) and instructed to respond accurately and rapidly to the deviants by pressing a button (spacebar). Before the behavioral test, they had a training session to familiarize themselves with the task. The training lasted until the subject's response accuracy reached 80%, which usually took 10–15 minutes. The time window for an acceptable response was set as 200–1000 ms after the onset of stimulus. The subject attended to the same stimuli as those presented in the EEG experiment. Reactions to violations of the auditory rule were assessed on the preattentive levels using latencies of MMN and on attentive levels using RT. For each subject, two blocks were presented (100 presentations per deviant type) and RT to each type of deviant was recorded.

### Data recording and analysis

An ESI-128 system (Neuroscan, Sterling, VA, U.S.) was used and the ERPs were recorded from the scalp with a electrocap carrying 64 Ag/AgCl electrodes placed at standard locations (the extended international 10–20 system) and two mastoids (LM and RM). The electroocular activity was recorded with electrodes attached to the infraorbital ridge and on the outer canthus of the left eye. The reference electrode was attached to the tip of the nose, and the ground electrode was placed on the forehead. Electrode impedances were kept<5k Ohm. Alternating current signals were filtered on-line with a band-pass of 0.5–100 Hz and sampled at a rate of 500 Hz. The recording data were band-pass filtered (1–25 Hz) off-line with a finite impulse response filter. Epochs of 600 ms time window, starting 100 ms before the onset of stimulus were obtained from the continuous data and rejected when fluctuations in amplitude>75 µV. The ERPs evoked by standard and deviant stimuli were calculated by averaging individual trials within a 600 ms time window, including a 100 ms prestimulus baseline (excluding the standard that immediately followed a deviant). MMN was derived by subtracting the ERP response to the standard from that to the deviant stimuli. For ERP quantification, individual amplitudes were computed and sent for statistical analysis. For statistical assessment of possible anterior-posterior and lateralization effects, we conducted repeated measures analyses of variance (ANOVA) with lexical tone (two levels: T2 vs. T4), sagittal electrode site (three levels: frontal, central, parietal), and lateral electrode site (three levels: left, middle, right) as the intra-subject factors, followed by post-hoc testing performed using the Tukey's test. The Greenhouse-Geisser adjustment was applied when the variance sphericity assumption was not satisfied. To estimate the neural sources of the MMN responses to violation of the auditory rule, a dipole analysis was performed for each subject (Curry software, Neuroscan). Because the digitized positions of the electrodes used in this experiment were not available, the source label-matching algorithm was used. This algorithm was based on a model of the extended international 10–20 system. The dipole was analyzed by applying a rotating dipole model (mirror dipoles, seed points were set for all of the participants in the same location in the bilateral auditory cortex (superior temporal gyri)) [48], with maximal distance from the seed points was 30 mm, and the minimal distance between two dipoles was 90 mm) [49]. This model localized two symmetrical sources in the two hemispheres using a standard boundary element model (BEM). First, dipole localization was performed within a time window from 150 to 350 ms after the onset of stimulus. Second, for each subject, the time point with the best explanation of variance was chosen for repeated dipole localization. Average dipole strengths were obtained from individual subjects, for each hemisphere, and for each deviant condition.

## Supporting Information

Figure S1 **Grand average of mismatch negativity (MMN) recorded at the fronto-central, central, parietal and mastoid sites.** (A) MMN responses to the rising deviant type (T2). (B) MMN responses to the falling deviant type (T4). Note that there was a polarity reversal when recorded from the left and right mastoid sites (LM and RM).
(EPS)

Figure S2 **Dipole solutions.** The grand-average dipole solutions of MMN using the standardized brain's magnetic-resonance image (Curry-Warped brain). (Data from one subject was excluded from dipole analysis due to insufficient SNR).
(EPS)
